# Transaction of the Illinois State Dental Society for 1877

**Published:** 1877-10

**Authors:** 


*Transactions of the Illinois State Dental Society for* 1877.

This volume contains several interesting and valuable papers. We commend the name "Soc ety" for all State organizations: let the national ones only be styled "Associations."

